# Genomes of eight cultured microbes from soil sites in Wellesley, MA

**DOI:** 10.1128/mra.01174-25

**Published:** 2026-01-30

**Authors:** Mia Chao, Elizabeth Chou, Victoria Tang, Arla Hofer, Oumayma Dakhama, Shea Gardner, Vaibhavi James, Priya Patel, Sally Song, Clara Tessier, Annika Wolberg, Steven J. Biller

**Affiliations:** 1 Department of Biological Sciences, Wellesley College 8456, Wellesley, Massachusetts, USA; University of Strathclyde, Glasgow, United Kingdom

**Keywords:** soil

## Abstract

We present draft-quality genomes of eight cultured microbes isolated from two soil sites with different levels of leaf cover in Wellesley, MA. This dataset includes representatives of taxa including *Pedobacter*, *Bacillus*, *Paenibacillus*, *Streptomyces*, and *Flavobacterium*.

## ANNOUNCEMENT

Soil microbial communities are critical to the health, productivity, and functionality of terrestrial ecosystems through their contributions to biogeochemical cycling and interactions with plants (1, 2). Understanding soil microbial diversity can inform sustainable agricultural practices, provide insights into the functional roles of microbial groups, and aid in managing environmental health (3, 4). Leaf litter affects microbial nutrient cycling by adding nutrients to soils, thereby influencing microbial metabolism and shaping community composition (5). To expand our understanding of soil microbial genomic diversity and explore the impact of leaf litter cover, we sequenced a set of microbes isolated from the suburban campus of Wellesley College (Wellesley, MA, USA). Microbes were sampled from two sites near the Munger residence hall (42° 17′ 43″ N, 71° 18′ 22″ W). One site had exposed, compacted soil associated with its presence along a commonly used footpath, while the other site (approximately 5 m away) was less impacted and covered with ~15 cm of oak leaf cover. Approximately 100 g of surface soil was collected from each of the two locations. Replicate ~2 g subsamples of soil were mixed with sterile PBS buffer, and then the diluted cell suspensions were spread onto R2A agar plates (6) and grown at 30°C for three days. Individual colonies were purified by re-streaking for single colonies twice on fresh R2A plates and grown as before.

Four colonies from each of the two sampling sites were randomly chosen for sequencing. These isolates were grown overnight at 30°C in liquid R2A media with shaking (250 rpm). Genomic DNA was extracted from cells pelleted from 2 mL of culture using the NEB Monarch Genomic DNA extraction kit, yielding 4–30 µg of DNA as measured with the Qubit 1X dsDNA High Sensitivity kit (Invitrogen). Total genomic DNA libraries for nanopore sequencing were prepared without size selection using the Oxford Nanopore rapid sequencing DNA V14 Barcoding Kit (SQK-RBK114.24). The pooled libraries were then loaded into an Oxford Nanopore MinION R10.4.1 flow cell and sequenced using a Mk1B sequencer for 48 h. Raw Nanopore data were processed using Dorado 0.9.1 (https://github.com/nanoporetech/dorado) in SUP mode, generating 65,133–1,218,550 reads per sample (Table 1). All extraction and library preparation protocols were carried out following the manufacturers’ instructions.

**TABLE 1:** Genome assembly summary data

Strain ID	Site	Total reads	Read N50 (bp)	Total sequence data (bp)	Total contigs	Plasmid contigs	Assembly length (bp)	Assembly GC (%)	Assembly N50 (bp)	Nanopore coverage (×)	GTDB taxonomy (genus)	CheckM completeness (%)	CheckM contamination (%)	Predicted genes (total)	Assembly accession	SRA raw data accession
WC2501	Leaf covered	632,068	6,142	1,575,797,205	1	0	6,241,392	38.4	6,241,392	252	Pedobacter	98.09	0.66	5,198	GCA_052682415.1	SRR35310660
WC2502	Exposed	621,153	8,525	2,025,664,145	2	0	3,737,437	41.7	3,723,327	542	Bacillus	99.59	0	3,825	GCA_052682445.1	SRR35310659
WC2503	Exposed	1,051,143	7,740	3,367,740,353	2	1	5,629,584	35.3	5,317,538	598	Bacillus_A	99.43	0.31	5,752	GCA_052682895.1	SRR35310658
WC2504	Exposed	185,637	12,439	919,682,998	1	0	8,179,892	46.0	8,179,892	112	Paenibacillus_E	99.19	1.52	7,485	GCA_052682475.1	SRR35310657
WC2507	Leaf covered	65,133	10,755	200,034,471	3	1	5,789,317	35.2	5,339,450	35	Bacillus	99.43	0.22	6,011	GCA_052656125.1	SRR35310656
WC2508	Leaf covered	352,818	6,487	882,731,881	4	2	9,489,572	70.8	7,519,079	93	Streptomyces	99.88	1.86	8,391	GCA_052656385.1	SRR35310655
WC2509	Leaf covered	1,218,550	8,121	4,048,993,377	1	0	4,508,346	33.1	4,508,346	898	Flavobacterium	98.94	1.06	3,737	GCA_052683105.1	SRR35310654
WC2510	Exposed	191,642	5,477	313,389,403	3	1	3,721,180	41.7	3,685,107	84	Bacillus	99.59	0	3,817	GCA_052656105.1	SRR35310653

Genome assembly steps were performed using KBase (7), and default parameters were used for all software tools. The FASTQ format sequence data generated by Dorado were first filtered using Filtlong v0.2.1 (https://github.com/rrwick/Filtlong) and then assembled with Flye v2.9.4 (8). Assembly quality was assessed using the CheckM v1.1.2 lineage-specific workflow (9). geNomad v1.8.0 was used to identify putative plasmids in the assemblies (10). Taxonomic classifications were conducted using GTDB-Tk v1.7.0 (11). Genomes were annotated by NCBI using the NCBI Prokaryotic Genome Annotation Pipeline v6.10 (12).

We obtained eight high-quality draft genome assemblies, ranging between 3.73–9.49 Mbp in length and 33–71%GC (Table 1). All sequences are estimated to be >98% complete and contain <1.9% contamination. Genomes were identified as members of the genera *Pedobacter*, *Bacillus*, *Paenibacillus*, *Streptomyces*, and *Flavobacterium*.

## Data Availability

All sequence data are available under NCBI BioProject PRJNA1321493 and via the specific accession numbers listed in Table 1. Data analyses can be accessed through KBase via the overview narrative at https://doi.org/10.25982/229295.9/2589424

## References

[1] Hayat R, Ali S, Amara U, Khalid R, Ahmed I. 2010. Soil beneficial bacteria and their role in plant growth promotion: a review. Ann Microbiol 60:579–598. doi:10.1007/s13213-010-0117-1

[2] Keck F, Peller T, Alther R, Barouillet C, Blackman R, Capo E, Chonova T, Couton M, Fehlinger L, Kirschner D, Knüsel M, Muneret L, Oester R, Tapolczai K, Zhang H, Altermatt F. 2025. The global human impact on biodiversity. Nature 641:395–400. doi:10.1038/s41586-025-08752-2 40140566 PMC12058524

[3] Miransari M. 2011. Soil microbes and plant fertilization. Appl Microbiol Biotechnol 92:875–885. doi:10.1007/s00253-011-3521-y 21989562

[4] Fierer N. 2017. Embracing the unknown: disentangling the complexities of the soil microbiome. Nat Rev Microbiol 15:579–590. doi:10.1038/nrmicro.2017.87 28824177

[5] Song Y, Yu Y, Li Y, Du M. 2023. Leaf litter chemistry and its effects on soil microorganisms in different ages of Zanthoxylum planispinum var. Dintanensis. BMC Plant Biol 23:262. doi:10.1186/s12870-023-04274-z 37198548 PMC10193752

[6] Reasoner DJ, Geldreich EE. 1985. A new medium for the enumeration and subculture of bacteria from potable water. Appl Environ Microbiol 49:1–7. doi:10.1128/aem.49.1.1-7.1985 3883894 PMC238333

[7] Arkin AP, Cottingham RW, Henry CS, Harris NL, Stevens RL, Maslov S, Dehal P, Ware D, Perez F, Canon S, et al.. 2018. KBase: The United States Department of Energy Systems Biology Knowledgebase. Nat Biotechnol 36:566–569. doi:10.1038/nbt.4163 29979655 PMC6870991

[8] Kolmogorov M, Yuan J, Lin Y, Pevzner PA. 2019. Assembly of long, error-prone reads using repeat graphs. Nat Biotechnol 37:540–546. doi:10.1038/s41587-019-0072-8 30936562

[9] Parks DH, Imelfort M, Skennerton CT, Hugenholtz P, Tyson GW. 2015. CheckM: assessing the quality of microbial genomes recovered from isolates, single cells, and metagenomes. Genome Res 25:1043–1055. doi:10.1101/gr.186072.114 25977477 PMC4484387

[10] Camargo AP, Roux S, Schulz F, Babinski M, Xu Y, Hu B, Chain PSG, Nayfach S, Kyrpides NC. 2024. Identification of mobile genetic elements with geNomad. Nat Biotechnol 42:1303–1312. doi:10.1038/s41587-023-01953-y 37735266 PMC11324519

[11] Chaumeil P-A, Mussig AJ, Hugenholtz P, Parks DH. 2020. GTDB-Tk: a toolkit to classify genomes with the Genome Taxonomy Database. Bioinformatics 36:1925–1927. doi:10.1093/bioinformatics/btz848 PMC7703759 31730192

[12] Tatusova T, DiCuccio M, Badretdin A, Chetvernin V, Nawrocki EP, Zaslavsky L, Lomsadze A, Pruitt KD, Borodovsky M, Ostell J. 2016. NCBI prokaryotic genome annotation pipeline. Nucleic Acids Res 44:6614–6624. doi:10.1093/nar/gkw569 27342282 PMC5001611

